# Epidemiology, Risk Factors and Mortality of Fungal Bloodstream Infection: 14 Years of Experience at a Teaching Hospital

**DOI:** 10.1007/s11046-025-00965-3

**Published:** 2025-06-17

**Authors:** Júlia Pongrácz, Tamás Szabó, Emese Juhász, Miklós Iván, Katalin Kristóf

**Affiliations:** 1 Department of Laboratory Medicine, Semmelweis University, Budapest, Hungary; 2 Markusovszky Teaching Hospital, Szombathely, Hungary

**Keywords:** Bloodstream infection, Candidaemia, Invasive fungal infection, Epidemiology

## Abstract

Fungal bloodstream infection is a severe condition associated with high mortality rates. Major risk factors of fungal bloodstream infection (BSI) include immunosuppression, abdominal surgery and malignancy. The epidemiology and underlying morbidities vary by geographic region and institute. We analyzed the species distribution, antifungal susceptibility, risk factors and mortality of fungal bloodstream infection at our institute over a 14-year period. Four-hundred and fourteen episodes were detected, and the average annual incidence was 0.23/1000 admissions. The most frequent species was *Candida albicans* (54%), followed by *C. glabrata* (16%). Abdominal surgery (48.3%), a solid tumor (31.4%), and immunosuppression (26.3%) were identified as major risk factors. Abdominal surgery was more prevalent in patients suffering from BSI caused by *C. albicans* or *C. glabrata*. *C. tropicalis* and *C. albicans* were associated with increased mortality in patients with neutropenia and/or immunosuppression. Azole prophylaxis in patients suffering from a hematologic malignancy or who recently had undergone organ transplantation increased the risk of *C. krusei* BSI. Overall 30-day mortality was 45.7% in any fungal BSI. Mortality of *C. tropicalis* BSI (59.4%) was the highest. Acquired antifungal resistance was not detected during the study period.

## Introduction

The prevalence of invasive fungal infections has increased over the past decades, as a result of a growing patient population receiving immunosuppressive treatments and advancements in critical care. Fungi are ranked as the ninth most frequent pathogens causing bloodstream infection (BSI), detected in 3.1% of BSI [1]. *Candida* spp. and among them *C. albicans*, are the most frequent fungal bloodstream isolates. Incidence and species distribution varies from region to region, and a shift to non-*albicans* species has been reported [2–4]. Isolates with acquired resistance to one or more groups of antifungals have been detected [5].

The mortality of fungal BSI is high, ranging from 27.7% [4] to 52% [6, 7] reported in various studies. Established risk factors of fungal BSI include immunosuppression, diabetes mellitus (DM), malignancy, the presence of intravenous devices, total parenteral nutrition (TPN), broad-spectrum antibiotic use, abdominal surgery and prior fungal colonization. The identification of patient populations at risk is indispensable in order to diagnose and treat fungal BSI in a timely manner. The knowledge of local data is a useful tool in the selection of empirical antifungal agents.

The goal of our study was to collect local data on the epidemiology of fungal BSI at our institute, Semmelweis University, the largest healthcare provider of the central region of Hungary, providing services for up to 3 million people. During assessment of our results, we also determined if there is any increase in the prevalence of non-*albicans* species or strains with acquired resistance mechanisms; also, if any patient demographics or underlying conditions are related with an increased risk of morbidity or mortality of fungal BSI.

## Materials and Methods

Data was collected retrospectively from all episodes of fungal BSI in adult patients treated at Semmelweis University, Hungary, a 2250-bed teaching hospital, from January 1, 2010 to December 31, 2023. An episode of fungal BSI was defined as the isolation of at least one fungal species from blood culture. Episodes were considered separate if they occurred at least 30 days apart. Polyfungal episodes (positive blood cultures with more than one identified fungal species) were considered as a group distinct from monofungal BSIs. Concomitant bacterial growth was also recorded. Patient demographic data, underlying diseases, comorbidities (DM, organ failure, malignancy, immunosuppression), risk factors of fungal BSI (e.g. presence of intravascular catheters, mechanical ventilation, TPN), and isolate characteristics (species and antifungal susceptibility) were recorded along with prior colonization and blood culture time-to-positivity (TTP). The administration of parenteral antifungal medication in the 30 days preceding the positive blood culture was noted. Treatment with third or fourth generation cephalosporins, piperacillin-tazobactam, carbapenems or aminoglycosides during the 30-days preceding fungal BSI was defined as broad spectrum antibiotic therapy.

Blood samples were processed using BacT/ALERT 3D automated blood culture system until 2021 and the BacT/ALERT Virtuo system from 2021, using FA Plus blood culture bottles (BioMérieux, Marcy-l’Étoile, France). Isolates were identified by either carbohydrate assimilation profiles (API 20C Aux yeast identification system, BioMérieux, Marcy-l’Étoile, France) and morphology of pseudohyphae on malt agar (2010–2012) or by matrix-assisted laser desorption ionization-time of flight mass spectrometry (MALDI-TOF/MS, Bruker Daltonik GmbH, Germany, from 2012). Isolates collected between 2010 and 2012 were stored and identification was verified by the MALDI-TOF method. Candida isolates are referred to by their anamorph name in the article. Routine antifungal susceptibility testing was performed with E-test® (BioMérieux) or the Sensititre YeastOne panel (TREK Diagnostic Systems, United Kingdom). MIC results were interpreted according to current EUCAST clinical breakpoints.

The statistical software suite R (version 4.3) was used for data analysis [8]. Statistical association between the existence of risk factors and detection of a fungal species in BSI was assessed based on the relative risk of an infection with or without the presence of the risk factor(s). The primary endpoint informing on the prognosis and severity of BSI was the 30-day survival of patients after blood culture positivity. Survival of patients stratified by predisposing factor and predominant species was compared based on the hazard ratio of Cox-regression models calculated with the ‘R’ survival package [9]. Age at diagnosis was included as an additional explanatory variable to correct for the possible bias introduced by the slight differences in the median age of patients in strata. In addition to the main model, separate models were fitted to male and female patient populations, accounting for possible gender-related biases.

## Results

**Fig. 1:**
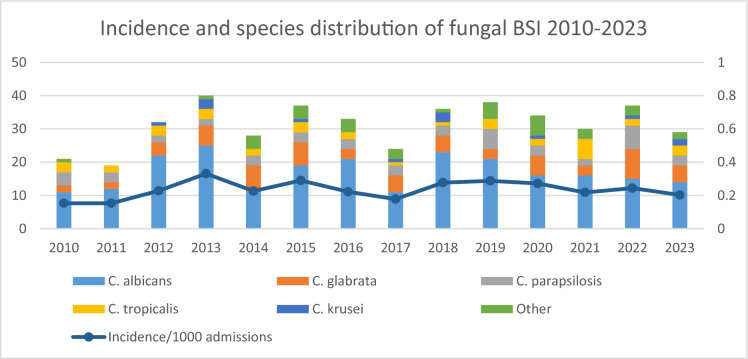
Incidence and species distribution of fungal BSI at Semmelweis University, 2010–2023

A total number of 414 episodes of fungal BSI was detected over the 14-year study period. The incidence of fungal BSI was quite stable during the study period; the average annual incidence was 0.23/1000 admissions; the annual incidence was the lowest in 2010 (0.15/1000 admissions) and highest in 2013 (0.33/1000 admissions) (Fig. 1). During the assessed period, the rate of fungal BSI was 3.4% among all positive blood cultures.

The predominant species was *C. albicans* (54%), followed by *C. glabrata* (16%), *C. parapsilosis* (11%), and *C. tropicalis* (8%). Other, less frequently isolated species included *C. krusei* (n = 13), *Saccharomyces cerevisiae* (n = 8),* C. lusitaniae* (n = 7), *C. kefyr* (n = 5), *C. fabianii* (n = 4), *Ogataea polymorpha* (n = 1), *Pichia cactophila* (n = 2), *C. utilis* (n = 1), *C. guilliermondii* (n = 1), *Trichosporon asahii* (n = 2), *Magnusiomyces capitatus* (n = 3), and *Magnusiomyces clavatus* (n = 1). Twenty-three blood cultures were polyfungal (5.5% of all fungal BSI episodes), 2 or 3 different species were cultured. *C. albicans* was detected in 78.3% (n = 18) and *C. glabrata* was detected in 65.2% (n = 15) of polyfungal cultures. The most common combination was *C. albicans* and *C. glabrata* (10 of 23 episodes, 43.5%).

Concomitant bacterial growth was detected in 21.6% (n = 88) of fungal BSI episodes. Gram-positive bacteria, Gram-negative bacteria and mixed (Gram-positive and Gram-negative bacterial growth) were isolated in 14.7%, 3.6% and 2.9% of all episodes, respectively. The most common bacteria were coagulase-negative *Staphylococcus* spp. (n = 35), *Enterococcus faecium* (n = 21), *Enterococcus faecalis* (n = 12), Enterobacterales spp. (n = 12), *Pseudomonas aeruginosa* (n = 8), other non-fermenting Gram-negative species (n = 9), *Staphylococcus aureus* (n = 6), *Lactobacillus* spp. (n = 6) and alpha-haemolyzing streptococci (n = 2). The 30-day mortality of BSI involving fungi and bacteria was 40.9%, similar to the overall mortality rate.

Some fluctuations were noted during the years assessed, but species distribution was stable during the study period. There was no significant shift toward non-*albicans* species. Overall TTP of the blood culture bottles was 1.4 days. Blood culture TTP was longer for *C. glabrata* than for *C. albicans* and *C. parapsilosis* complex (in average, 2.08, 1.34 and 1.26 days, respectively). BSI caused by *C. tropicalis* and *C. krusei* were detected earlier compared to other species (0.71 days and 0.78 days, respectively). All isolates were wild type organisms; no acquired antifungal resistance was detected.

**Fig. 2:**
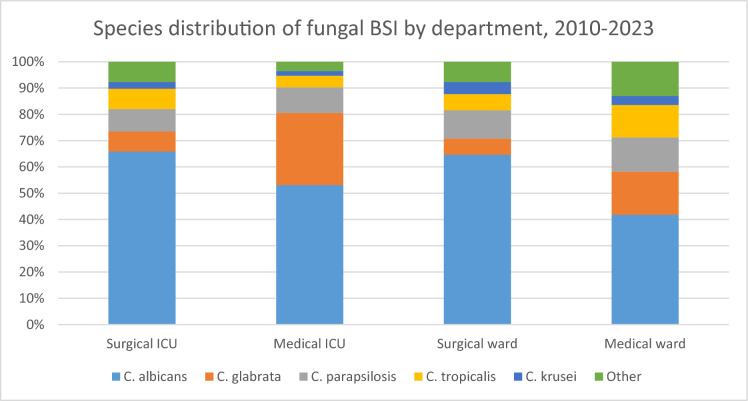
Fungal BSI species distribution in the ICU and non-ICU departments

*C. albicans* was the predominant species in the intensive care units (ICUs) (60.35% of isolates), while non-*albicans* fungal species were more prevalent in non-ICU departments (51.18%). The proportion of *C. glabrata* was higher in the ICU (17.62%) than the non-ICU setting (13.27%) (Fig. 2).

The number of male patients was 1.56 times higher than the number of female patients in the study (60.9% of patients were male, while 39.1% were female). The average age of the patients was 63.57 years, with no significant difference between men and women (63.97 and 62.94 years, respectively). Over half the patients (51.93%) were treated at surgical and medical ICUs (14.5% and 37.43%, respectively). Non-ICU patients with fungal BSI (48.07%) were treated at Internal Medicine Departments or surgical wards (32.61% and 15.46%, respectively).

The most frequent comorbidities in the 30 days before the positive BSI result were abdominal surgery, a solid tumor, and immunosuppression (48.3%, 31.4% and 26.3%, respectively). DM and kidney failure were present in almost the third of patients (29.7% and 30.2%, respectively). Nearly 80% of patients with fungal BSI received broad spectrum antibiotic treatment. Antifungal treatment prior to detection of fungaemia was prominant in the *C. krusei* group, along with a hematologic malignancy, neutropenia, and organ transplantation (46.1%, 30.8%, 38.5% and 23%, respectively). Prior abdominal surgery was more frequent in the medical history of patients suffering from *C*. *albicans* and *C. glabrata* BSI (52.7% and 55.5%, respectively) compared with other species.

Table 1 presents the percentage of patients with the clinical characteristics listed above in case of each species of fungi.

**Table 1:** Clinical characteristics of patients with fungal BSI

	*C. albicans*	*C. tropicalis*	*C. glabrata*	*C. parapsilosis*	*C. krusei*	*Other candida *spp.	*Saccharomyces *spp.	Polyfungal	Total
Number of patients	220	32	54	39	13	17	9	23	414
Median age (range)	67 (21–92)	69.5 (47–90)	69 (24–86)	66 (22–88)	55 (37–64)	66 (46–90)	51 (23–68)	62 (23–86)	67 (61–92)
ICU stay	57.1%	38.9%	58,82%	44.68%	38.46%	30%	44.4%	34.7%	51.93%
Prior abdominal surgery	52.7%	37.5%	55.5%	41%	38.5%	41.2%	33%	39.1%	48.3%
Solid tumor	32%	28%	40%	25.6%	15.4%	23.5%	11.1%	39.1%	31.4%
Hematologic malignancy	7.7%	6.2%	9.3%	15.4%	30.8%	11.8%	11.1%	4.3%	10.1%
Neutropenia	8.2%	9.4%	1.8%	15.4%	38.5%	5.9%	11.1%	8.7%	9.9%
Immunosuppression	21.4%	21.9%	22.2%	41%	53.8%	41.2%	22.2%	30.4%	26.6%
Organ transplantation	1.4%	3.1%	3.7%	5.1%	23%	17.6%	11.1%	4.3%	4.1%
DM	29.5%	34.4%	33.3%	28.2%	23.1%	41.2%	11.1%	13%	28.7%
Kidney failure	35%	15.6%	22.2%	20.5%	23.1%	47%	33.3%	26.1%	29.9%
Liver failure	5.9%	9.4%	1.8%	0	0	23.5%	0	0	5.5%
Iv. catheter	95.9%	93.6%	96.3%	94.9%	92.3%	94.1%	77.8%	95.7%	95.7%
Mechanical ventilation	60%	46.9%	66.7%	38.5%	30.8%	58.8%	55.5%	39.1%	56.8%
Parenteral nutrition	33.6%	18.7%	24.1%	38.5%	53.8%	29.4%	11.1%	26.1%	30.7%
Immunosuppressive treatment	15%	12.5%	20.4%	25.6%	38.5%	29.4%	22.2%	21.7%	18.8%
Broad spectrum antibiotic treatment	81.4%	68.7%	81.5%	71.8%	69.2%	70.6%	77.8%	87%	78.7%
Prior antifungal treatment	5%	0	9.2%	7.7%	46.1%	0	0	4.3%	6.8%
30-day mortality	45%	59.4%	51.85%	38.5%	15.4%	52.9%	11%	56.5%	45.7%

Other Candida spp. = C. lusitaniae, K. marxianus, C. fabianii, P. cactophila, C. jadinii, and M. guilliermondii; Saccharomyces spp. = O. polymorpha and S. cerevisiae.

Statistical analysis was performed to check if any underlying condition or clinical feature predisposed to BSI with any given species of fungi. Men who had undergone abdominal surgery were more likely than patients without surgery to suffer from BSI caused by *C. albicans* (*p* = 0.02). This association was not present in women (*p* = 0.76). *C. albicans* and *C. tropicalis* BSI was associated with kidney insufficiency (*p* = 0.02 and *p* = 0.05 respectively). Immunosuppression predisposed to BSI with *C. albicans* and *C. parapsilosis* (*p* = 0.02 and *p* = 0.04, respectively). Hematologic malignancy and neutropenia was associated with *C. krusei* BSI (*p* = 0.05 and *p* = 0.01, respectively). A hematologic malignancy, but not neutropenia, also predisposed to BSI with *C. albicans* and other *Candida* spp. ((*p* = 0.05 and *p* = 0.05, respectively). Patients with *C. tropicalis* BSI were more likely to be treated at an ICU (*p* = 0.05). Parenteral nutrition, solid malignancy and diabetes mellitus did not predispose to BSI with any particular fungal species.

Our hospital protocol includes routine surveillance cultures (nose, throat, axillary, and perianal swabs) for the detection of multidrug-resistant microorganisms in the ICU and the Hematology Departments at the patients’ first admission, followed by weekly follow-up cultures. Surveillance cultures were not performed in all cases at other departments, and relevant clinical samples for the detection of the source of fungal BSI were not available in all cases. When prior growth of fungi from clinical and surveillance samples was assessed, the species causing BSI was detected in 256 cases (61.84% of episodes). A fungal species different from the one detected from the bloodstream was isolated in prior clinical samples in 15 episodes. Cultures, when available, identified the source of infection as an abdominal abscess, an iv. catheter, or a wound (9.9%, 14% and 7.2%, respectively). The lower respiratory tract was colonized by the same species causing BSI in 29.9% of episodes, and the same species was isolated from the urine in 22.2% of cases.

**Fig. 3:**
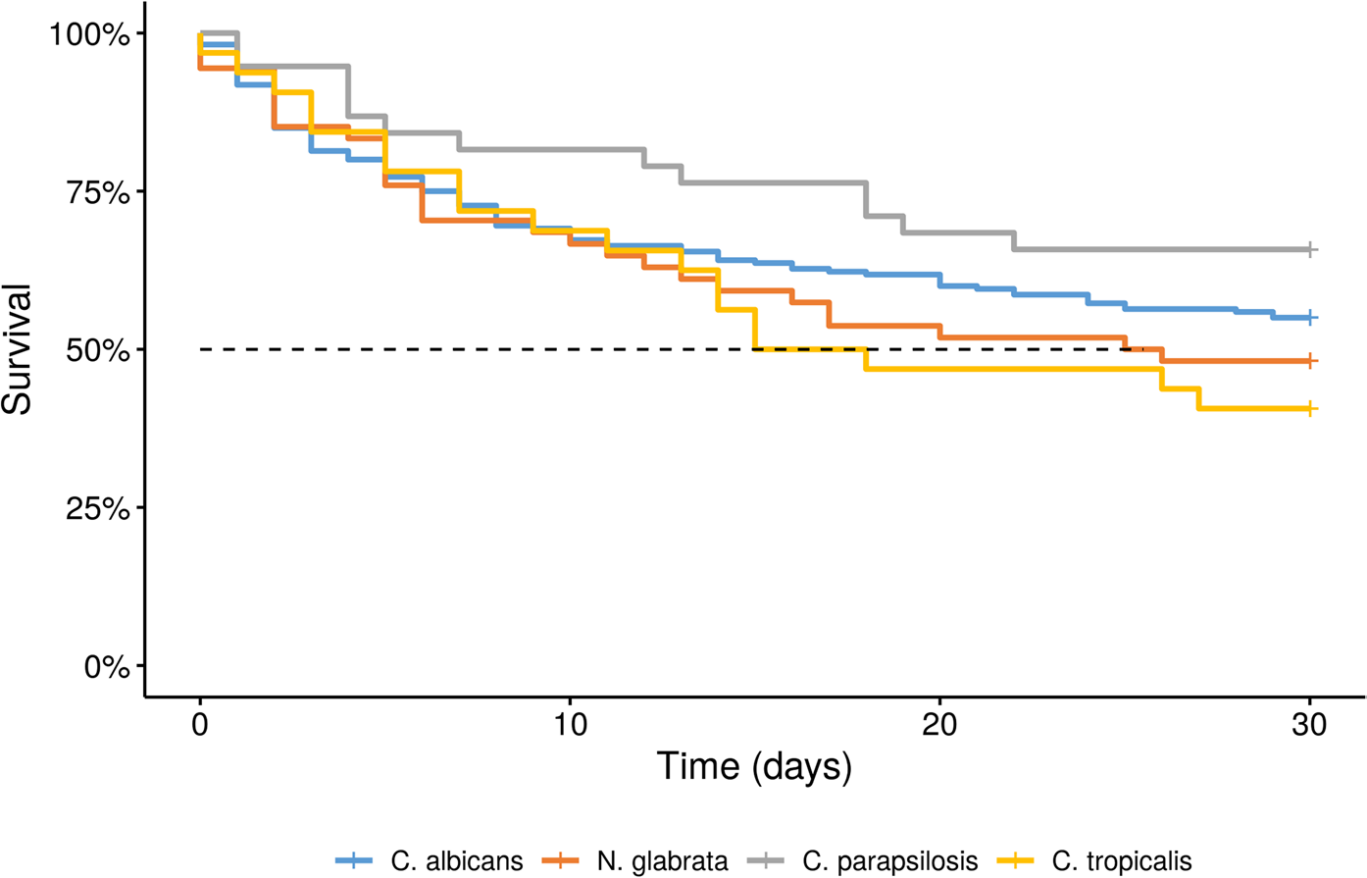
Kaplan–Meier curves for the four most common fungal species causing BSI

Overall 30-day mortality was 45.7%. BSI caused by *C. tropicalis* had the highest mortality rate (59.4%), while the mortality of *C. krusei* and *Saccharomyces* spp. were the lowest (15.4% and 11%, respectively) and the two patients with *C. neoformans* BSI both survived. Four cases of BSI caused by *Magnusiomyces* spp. (three cases of *M. capitatus* and one case of *M. clavatus*) were detected in the study: three patients were treated with a hematologic malignancy and were neutropenic at the time of the positive blood culture; two patients survived. Survival of the four most common fungal species is presented in Fig. 3. Mortality rates were compared if any given species could be linked to a significantly worse survival, and *C. tropicalis* BSI in female patients not treated at an ICU was associated with a significantly worse outcome (*p* = 0.04). There was no significant difference in mortality of the different species detected in the ICU.

Cox regression analysis was performed to compare mortality in patients suffering from BSI caused by different species (*C. albicans*, *C. glabrata*, *C. parapsilosis*, and *C. tropicalis*) suffering from different comorbidities. Major findings included that *C. tropicalis* was associated with increased mortality in patients with neutropenia; *C. albicans* was associated with worse outcomes in immunosuppressed patients and men receiving parenteral nutrition. There was no significant difference in the mortality of BSI caused by the four most prevalent species in patients whose medical history included abdominal surgery, a hematologic or solid malignancy, DM, or renal failure.

## Discussion

Since the symptoms of fungal BSI are not specific, diagnosis is challenging. Delayed initiation of proper antifungal treatment leads to worse outcomes in patients suffering from this high-mortality infection [10]. The aim of our study was to evaluate the local epidemiology of fungal BSI at our institute and identify any characteristics that may help identify the patient population at risk and guide initial empirical antifungal therapy.

The incidence of fungal BSI ranges from 0.2 to 3.7 cases/1000 admissions, depending on geographic location and center studied [11]. The incidence at our hospital (0.23 cases/1000 admissions) was a bit lower than incidence data reported from other European centers: 0.36/1000 admissions in Switzerland [12]; 0.48/1000 admissions in Ireland [13]; and 0.98/1000 admissions in Spain [3]. Over the past two decades, the proportion of *C. albicans* has decreased, and the proportion of non-albicans species (mainly *C. glabrata)* in the USA and Northern Europe; and *C. parapsilosis* in China, Latin America and Southern Europe) has increased around the world [11, 14]. The proportion of the most frequent species, *C. albicans* (54%)*,* remained relatively stable at our institute during the study period. However, the percentage of *C. albicans* bloodstream isolates was higher in the first five years of the study period compared to the last five years of the study period (63% vs. 48.8%, respectively), but the trend was not statistically significant [15]. The order of most frequent non-*albicans* species may vary from center to center even within the same geographic region. In our study, *C. glabrata* (16%) was the second most frequently detected species, while *C. parapsilosis* was the second most common species observed in studies at two other Hungarian centers; however, these studies both included pediatric patients, while our study did not, which could explain the difference [6, 16].

In our study, 5.5% of all fungal BSI episodes were polyfungal. In an 11-year single-center study from Sweden, mixed fungal infection was detected in 2.8% of candidaemia episodes, with *C. albicans* and *C. glabrata* being isolated the most frequently [17], similar to our findings. Polyfungal candidaemia has been associated with intravascular catheter use, TPN and gastrointestinal surgery [18]. In our study, the frequencies of risk factors in case of polyfungal candidaemia were not significantly different compared to monofungal candidaemia. Mortality of polyfungal BSI was 56.5%, which was higher than the average mortality rate of all monofungal BSIs (45%).

The pathogenicity of fungi differs by species: *C. albicans* and *C. tropicalis* are associated with higher mortality rates than low-virulence species such as *C. parapsilosis* and *C. krusei* [14]. In our study, *C. tropicalis* had the highest mortality rate (59.4%) among *Candida* spp., but the mortality of *C. glabrata* (51.85%) and rare Candida spp. (52.9%) were both higher than that of the more virulent *C. albicans* (45%), although not significantly. The higher rate of immunosuppression in the rare Candida spp. group may explain the higher mortality rate. Some form of immunosuppression was recorded more often in patients suffering from BSI caused by low-virulence or rare Candida spp. (*C. parapsilosis, C. krusei*, other Candida spp.), underlining this risk factor of invasive fungal disease.

Several studies have associated *C. tropicalis* BSI with hematologic malignancy [19], however, our results did not support these findings. A higher proportion of patients at our hospital suffering from a hematologic malignant disease or neutropenia was detected in the *C. krusei* group. *C. krusei* BSI has been linked to azole prophylaxis in patients with hematologic malignancies [20]; 6 patients out of 13 received azole treatment prior to *C. krusei* candidaemia in our study. Mortality *C. krusei* BSI was low (15.4%) at our center, opposed to the findings Schuster et al.: the 30-day mortality of *C. krusei* was 68% [21]; the relatively low sample size in both studies may account for the difference.

*C. glabrata* BSI has been linked with solid (mostly gastrointestinal) malignancy [22], older age and DM [23]. In our study, the proportion of patients with solid malignancy (40%) was numerically higher than in case of any other species, but statistically the difference was not significant. Our study did not find any underlying conditions to be significantly associated with *C. glabrata* BSI, supporting the results of Smyth et al. [24]

A higher ratio of patients developing BSI caused by rare *Candida* spp. suffered from liver failure (23.5%) compared with other species. Liver failure leads to impaired immune function due to phagocytic function damage, predisposing to invasive infection with a low virulence species [25].

The source of fungal BSI is usually the patients’ skin or gastrointestinal mucosa, where the fungi are present as part of the commensal flora. In case the gastrointestinal mucosal barrier is disrupted (e.g. chemotherapy, surgery), the fungus may invade the bloodstream. *C*. *albicans* and *C. glabrata* are frequent colonizers of the human gastrointestinal tract, explaining why abdominal surgery was more common in patients with BSI caused by these two species (52.7% and 55.5%, respectively).

Detection of fungal colonization at various body sites may predict an invasive infection. The Candida Colonization Index is a predictive score system defined as the ratio of the number of distinct body sites colonized by Candida spp. to the total number of body sites cultured. Its sensitivity in detecting invasive Candida infections is 75–80%, but its positive predictive value is low [26]. Systematic screening for fungi was not performed at our institute during the study period, but a review of the results of microbiological samples taken before the fungal BSI yielded the invasive fungus in more than half the cases, and the source of infection could be identified in 31.1% of cases. Since genotyping studies have confirmed that an invasive disease usually originates from a patient’s own colonizing fungal flora after skin or mucosal barrier impairment, detection of colonization can predict which fungal species may be involved in case of invasive infection [27].

Isolates harboring resistance to one or more antifungal agent have been detected around the world. The proportion of fluconazole non-susceptible isolates of *C. tropicalis* was 13.3% in Taiwan and 11.6% in China [28, 29]. Fluconazole-resistant *C. parapsilosis* strains were reported from Spain [30]. Echinocandin resistance typically develops during treatment in *C. glabrata* isolates. The rate of echinocandin resistant *C. glabrata* isolates is 2.3% in China [29] and 8–9.3% in USA centers [31], but resistant strains have been reported from Switzerland as well [32]. Acquired antifungal resistance of BSI isolates was not detected at our institute.

In conclusion, our study describes the epidemiology of fungal BSI at a single center over 14 years. The most frequently isolated species was *C. albicans* (54%), followed by *C. glabrata* (16%) and *C. parapsilosis* (11%). *C. tropicalis* and *C. albicans* were associated with increased mortality in patients with neutropenia or immunosuppression. Certain patient risk factors can be associated with BSI by a certain species. Abdominal surgery was more prevalent in patients suffering from BSI caused by *C. albicans* or *C. glabrata.* At our institute, patients with a hematologic malignancy, recent organ transplantation or neutropenia were more likely to suffer from BSI caused by *C. krusei*, in association with azole prophylaxis in this patient group.

The limitations of this study are related to its retrospective nature. Data was limited or lacking regarding the source of fungal BSI and the choice of antifungal treatment. Our study is limited to a single center, thus not representing regional data on a larger scale. The strength of our study is that it summarizes a long, 14-year time interval, and although it is a single-center study, our institute is one of the largest healthcare service providers in Hungary, offering patient care in all medical fields at the highest level of progressivity, including hematology departments and solid organ transplantation.

Although fungal BSI is uncommon, comprising approximately 3% of all positive blood cultures, it is associated with high mortality rates (45.7% in our study), and should be suspected in patients who had undergone abdominal surgery, suffer from a malignant disease, have kidney insufficiency or immunosuppression, and appropriate diagnostic evaluation must be performed. Growth of fungi from prior surveillance samples may aid in identifying patients at risk and be the basis of empirical treatment, since acquired antifungal resistance was not detected at our institute during the study period.
